# Preference-Aware User Access Control Policy in Internet of Things

**DOI:** 10.3390/s23135989

**Published:** 2023-06-28

**Authors:** Songnong Li, Yao Yan, Yongliang Ji, Wenxin Peng, Lingyun Wan, Puning Zhang

**Affiliations:** 1 State Grid Chongqing Electric Power Research Institute, Chongqing 401123, China; 2 School of Communication and Information Engineering, Chongqing University of Posts and Telecommunications, Chongqing 400065, China

**Keywords:** Internet of Things, user access, preference aware, load balancing, network slicing

## Abstract

There are multiple types of services in the Internet of Things, and existing access control methods do not consider situations wherein the same types of services have multiple access options. In order to ensure the QoS quality of user access and realize the reasonable utilization of Internet of Things network resources, it is necessary to consider the characteristics of different services to design applicable access control strategies. In this paper, a preference-aware user access control strategy in slices is proposed, which can increase the number of users in the system while balancing slice resource utilization. First, we establish the user QoS model and slice QoS index range according to the delay, rate and reliability requirements, and we select users with multiple access options. Secondly, a user preference matrix is established according to the user QoS requirements and the slice QoS index range. Finally, a preference matrix of the slice is built according to the optimization objective, and access control decisions are made for users through the resource utilization state of the slice and the preference matrix. The verification results show that the proposed strategy not only balances slice resource utilization but also increases the number of users who can access the system.

## 1. Introduction

In recent years, the gradual popularization of mobile intelligent terminals of the Internet of Things and the development of emerging business demands such as smart cities, telemedicine, unmanned driving, and virtual reality have put forward stricter requirements for the Internet of Things [1]. For example, there are fault diagnosis applications in industrial Internet of Things applications. The authors of [2] proposed an unsupervised cross-domain rolling-bearing fault diagnosis method based on time–frequency information fusion to enhance the diagnosis accuracy and strong robustness in the industrial Internet of Things. The authors of [3] designed a feedback-aided PD-type ILC design for time-varying systems with non-uniform trial lengths, which can achieve asymptotic tracking of the desired trajectories for time-varying systems with non-uniform trial lengths in the industrial Internet of Things. Different services have different network requirements. According to different service requirements, the network can be divided into three application scenarios: enhanced mobile broadband (eMBB), ultra-reliable and low-latency communication (URLLC) and massive machine-type communication (mMTC) [4]. These three different scenarios correspond to different Quality of Service (QoS) requirements. The eMBB type of traffic focuses on services that require large and guaranteed bandwidth, such as augmented reality services; the uRLLC type mainly serves low-latency, high-reliability services, such as the Industrial Internet of Things. Finally, mMTC needs to support large-scale devices that can tolerate certain access delays, such as smart-home services. The business requests of the Internet of Things may not only belong to one type of business; for example, the requests related to smart medical care belong to both URLLC business and mMTC business. Therefore, there are situations where a service has multiple access options. Therefore, in order to connect users to the network as much as possible while ensuring the QoS requirements of users, it is necessary to perform appropriate access control on users. Network slicing can provide a logically separated independent network, which can realize QoS isolation of different traffic and support more service requests. Due to the introduction of network slicing technology, in IoT network slicing, user access is fundamentally different from that in traditional mobile networks [5]. On the one hand, network slicing is logical virtualization and isolation by sharing physical infrastructure. Therefore, physical and virtual resources should be considered in network slicing resource allocation. On the other hand, in order to meet service requirements, user equipment needs to select an appropriate network slice for access. Due to resource constraints, not all base stations can provide specific slice services. For users with specific QoS requirements, the association between network slices and base stations should be jointly optimized. In IoT access network slicing, the service guarantee for slice users is more complicated than that in traditional mobile networks because not only are physical access constraints between users and base stations required, but logical access constraints between users and slices also need to be considered. Therefore, the user–base station–slicing three-layer association problem has become an important and challenging problem, and an appropriate user access control strategy is the key to solving this problem.

There have been studies for user access control in slicing. The authors of [6] studied access control in slicing, considered the delayed access problem, modeled the problem as a multiple knapsack problem with random reach for unknown future information, and proposed a heuristic algorithm to obtain suboptimal gains under resource constraints. The authors of [7] guaranteed the minimum data rate requirement of users within a slice by controlling the number of users accessing the slice and proposed a user access control policy that rejected new users when the slice could not guarantee the rate requirement of all users and removed users when a tenant changed its resource allocation policy and could not meet the user rate; they described the user access problem as a network slicing game problem by solving the Nash equilibrium to improve network performance. The authors of [8] designed a joint resource allocation and admission control scheme to maximize the number of terminals that could be accessed by slicing while satisfying the terminal interference constraint. In [9], an access control scheme considering inter-slice and intra-slice priorities was proposed for network slicing, which improved user experience while increasing throughput and resource utilization. The authors of [10] pointed out that admission control and resource allocation mechanisms are pivotal for realizing network slicing efficiently; this paper proposed an approach encompassing intelligent and efficient mechanisms for admission control and resource allocation for network slicing in the 5G core network. The admission control mechanism introduces two solutions, one based on reinforcement learning and the other based on deep reinforcement learning, considering the QoS requirements of 5G use-cases, differentiating network core nodes from edge nodes and processing slice requests in time windows to favor the service provider’s profit and resource utilization. Reference [11] presented a Network Slice Selection Function (NSSF) validation for IoT scenarios in an E2E network slicing architecture, considering traffic prioritization for critical applications. For this, data analytics, machine learning and multi-criteria decision-making methods were used. However, the above-mentioned references fail to consider the problem of multiple access control for a single service, which can lead to load imbalance between slices and thus affect the network utility.

**Figure 1:**
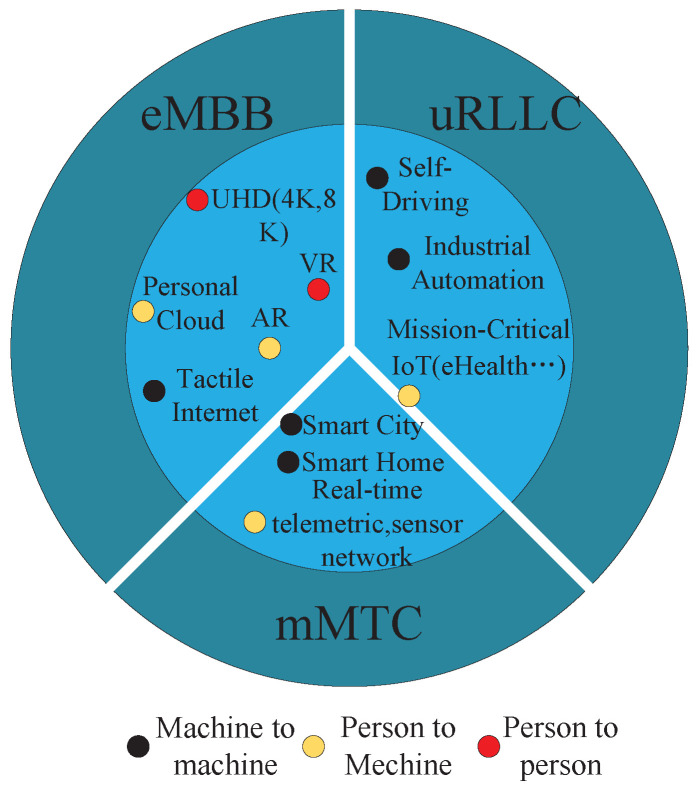
Network slicing service distribution diagram.

There is similarity in QoS requests for different types of services. As presented in Figure 1, only a small number of applications can access only one slice type, while the performance index requirements of most applications are located between two or more slices, and there are multiple access options. For example, the data rate of a user can be satisfied by multiple slices simultaneously [12], i.e., there will be an intersection of QoS between different slices. Therefore, in this paper, we consider the existence of such users in the IoT system and define such users as fuzzy users, and for users with multiple access options, reasonable user access is achieved by designing an access control mechanism. First, the fuzzy users are defined, and the users with multiple access options are filtered. Then, the user preference matrix is established according to user QoS requirements and slice QoS interval, the slice preference matrix is built according to the objective function, and fuzzy users and slices are grouped according to the preference matrix. Thus the main contributions of this paper are as follows.

- A fuzzy user selection strategy is proposed by considering the characteristics of different services with similar QoS requirements, the user’s delay, data rate and reliability indicators, and corresponding slice QoS intervals are designed. The slice membership function is defined to calculate the degree of membership of users to different slices, and the fuzzy users with multiple access options are screened out according to the degree of membership.
- A user access control strategy is designed, and user-slice grouping is performed according to the preference matrix by designing the slice preference matrix and the user’s preference matrix. Furthermore, user access control decisions are made according to the current resource utilization of different slices, and users are connected to slices with low resource utilization, thereby effectively increasing the number of access users while ensuring load balancing.

The remainder of this paper is organized as follows. Section 2 introduces the related work. Section 3 designs the system model. Section 4 proposes the user QoS demand analysis. Section 5 is problem modeling. Section 6 presents the user association strategy. Section 7 describes the simulation analysis. Section 8 concludes the paper.

## 2. Related Works

In Cloud Radio Access Network (C-RAN) for IoT, the available resources and the distance to the user vary from one Remote Radio Head (RRH) to another because the location of the user is uncertain. Therefore, there are differences in the QoS performance that various RRHs can provide for users. For each user, not all RRHs can provide the service. Therefore, choosing the right RRH for each user is a key issue.

The problem of user access control in IoT has been studied and analyzed by several researchers. The authors of [13] investigated the problem of associating users with RRHs to minimize the number of switches in the network while ensuring user QoS. A reinforcement learning algorithm was used to ensure that the user had a long communication connection after accessing the RRH. The index of candidate RRHs and the distance, angle and direction between the user and the RRH was considered as the state space, and an intelligent body selected the appropriate RRH for the user to associate with the candidate RRHs so that the cumulative reward could be maximized. In [14], a concept of coupled multiple access under ultra-dense networks was proposed to allow users to access multiple base stations for uplink and downlink, respectively. By constructing a decoupled multi-access matching game, a switching matching game algorithm was adopted to obtain higher data transmission rates with guaranteed user QoS requirements. The authors of [15] considered the user access problem in small-cell systems and proposed a Gale–Shapley stable matching algorithm based on user access control to optimize the throughput performance of each user by selecting the access point with the maximum channel gain.

Network slicing has been widely recognized as a key architectural technology for cellular IoT. In C-RAN slicing, the user access strategy is more complex due to the inclusion of network slicing. An efficient user access scheme for RAN slicing was proposed in [16] to improve network throughput while reducing equipment switching cost. User access control under wireless network slicing has a crucial role in load balancing, radio spectrum efficiency and network efficiency [17,18,19]. User access in a network slicing architecture is related to how users access the base station and also to the slices associated with the base station. Increasing the data rate of users in traffic intensive areas under traditional network architectures is limited by interference, network congestion, and operational cost. In [20], a new network slicing architecture is proposed to solve the user access problem in wireless LANs using technologies such as SDN (Software Defined Network) and NFV (Network Function Virtualization). Firstly, the isolation property of slices was used to eliminate the interference between base stations, while user access control algorithms were adopted to find a stable match between user devices and different network slices. The authors of [21] pointed out that user access based on the maximum signal-to-noise ratio was not an effective access control strategy. Therefore, the authors investigated the user access problem for multi-tenant network slices in heterogeneous networks combined with fairness, quality of service, energy consumption and energy efficiency aspects while considering the priority of tenants and users, and utilized genetic algorithms were implemented for user access control to maximize the weighting and rate.

Different services in the network have large variability in throughput and delay requirements, and to achieve different service requirements, the authors of [22] proposed a network slicing throughput and delay model, and the user access problem was abstracted as a mixed integer programming problem. For small networks, the global search method was used to solve user access decisions. For large networks, heuristic algorithms were adopted to balance the bandwidth and delay issues of different services so as to ensure the quality of user access. However, the above-mentioned references do not consider the access control problem for multiple access users in network slicing.

The above results ignore the multiple access control issues of a single service, which leads to unbalanced load among slices, thereby affecting network utility. However, this paper considers the existence of such users in the system, defines such users as fuzzy users, and realizes reasonable user access through an access policy designed for users with multiple access options. Therefore, the number of users connected to the system can increased while the load is relatively balanced.

## 3. System Model

**Figure 2:**
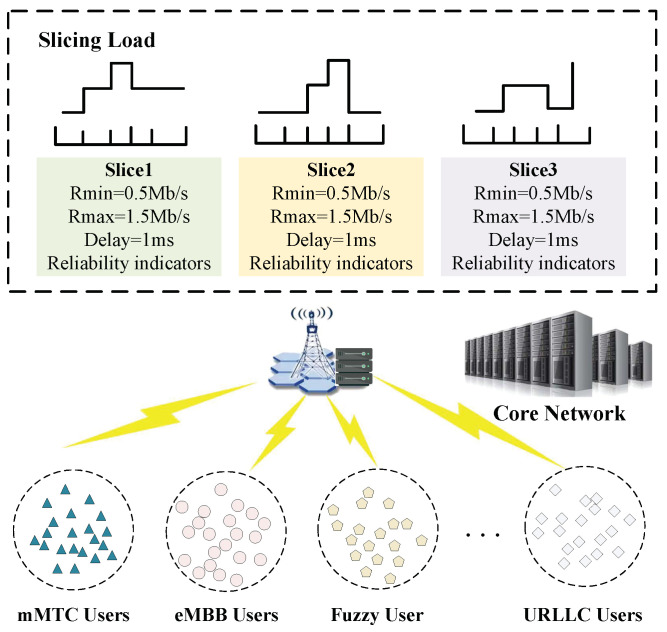
Access control schematic.

In this paper, the system model consists of an RRH, a Base Band Unit (BBU), slices of different service types and users in an IoT C-RAN network slice scenario. Figure 2 shows that there are three types of slices associated with the RRH and four types of user requests within the RRH coverage area, namely, enhanced mobile broadband (eMBB) users, ultra-reliable and low-latency communication (URLLC) users, massive machine-type communication (mMTC) users and a large number of fuzzy users. Fuzzy users are described specifically in Section 4. As shown in Figure 2, each slice has data rate, delay and reliability metrics, and when a usersubmits a business access request, the slice selects the user that can satisfy its QoS requirements for access. However, we must consider that slices have different service times for different tasks, i.e., some tasks have large service delays, and the occupied resources cannot be released in a short time. Therefore, there are two cases of high resource utilization and low resource utilization for different slices in the same period. Considering the different resource utilization of slices in different periods, to reduce the load imbalance of slices and ensure the system throughput, effective access control of slices is required.

Thus, the requests in the system can be represented as having a large number of fuzzy user requests and a small number of extreme user requests. In this paper, we assume that there are a large number of fuzzy users in the system, i.e., there are multiple slices in the system that can satisfy the QoS demand of those fuzzy users, but each user can only access one of the slices. First, the slices select the users that can be satisfied based on their QoS requirements. However, when the slice is relatively low in available resources, the user accesses the slice with relatively low resource utilization that can meet its QoS need.

Considering the whole system, in order to provide access to as many users as possible, reasonable access control for users is required; namely, the system must determine which slices can create the maximum number of users accessing the system while ensuring the relative load balance of slices in the system under the condition of ensuring the QoS requirements of users. The label of different types of slices is denoted by N={1,2,3,…,N}, the number of users in the system is defined as U={1,2,…,u}, and the user attribute characteristics are modeled as $t_{u}=\left(R_{u},\varepsilon_{u},T_{u}\right)$, where $R_{u}$ is the data rate request of users, $\varepsilon_{u}$ is the reliability requirement of users, and $T_{u}$ is the delay requirement of users.

## 4. QoS Demand Model

This section describes the delay, rate and reliability metrics of fuzzy users. First, the fuzzy users are defined; eMBB, URLLC, and mMTC are the three main types of services in 5G. In the 5G large-scale IoT scenario, some requests belong to both mMTC services and URLLC services: that is, the mMTC service and URLLC service have an intersection of QoS. Here, we consider users at the intersection of multiple service QoS as fuzzy users. For example, the QoS attribute interval of a slice $n_{1}$ is denoted as $\Omega_{1}$, the QoS attribute interval of slice $n_{2}$ is denoted as $\Omega_{2}$, and so on. Additionally, the intersection of attributes between slices is denoted as $\Delta =\Omega_{1}\cap \Omega_{2}\cap$…$\cap \Omega_{n}$. The QoS request of user *u* is denoted as $reqest_{u}$. Then, if Formula (1) is satisfied,

(1) $$request_{u}\in \Delta ,\exists u\in U,$$

user *u* is defined as a fuzzy user. If Equation (1) is not satisfied, then user *u* is grouped with other users, where Δ is denoted as the intersection part of QoS attributes between any two or more slices.

Assuming that the number of resources in different slices is represented as a set $M=\{M_{1},M_{2},\ldots ,M_{N}\}$. The user request is not served immediately and may even be denied due to the network state. In this case, the user will choose to re-initiate the request after a random delay or join the queue for queuing. To ensure fairness [23], this paper uses queuing to describe the delay when the user does not have immediate access to the network. Considering that an excessively long queue can lead to a long queuing delay—which causes some users to regret joining the queue or leave it, resulting in queue instability—this paper considers reducing the queue length as much as possible, which means it allows users to access other slices that can satisfy their QoS requests when the slice resources are limited.

We assume that the arrival of user requests obeys a Poisson process and that the time of user services is exponentially distributed, and, considering that users have multiple choices of access slices, we consider M/M/n queuing to model this process.

Assuming that user requests obey a Poisson distribution with parameter λ, the probability that the number of tasks generated in time interval *T* is defined by Formula (2).

(2) $$P\left(k\right)={\left(\lambda T\right)}^{k}e^{-\lambda T}/k!$$

where λ is the average number of tasks arriving per unit of time. The service time for each request obeys an exponential distribution, with parameter γ as

(3) P{t<t}=1−exp(−λt)

It is assumed that the arrival of the request and the receipt of the service by the user are independent of each other. We assume that $\eta_{u,n}$ denotes the service efficiency of user *u* on slice *n*. Thus, the service rate of the whole system is $\sum_{n}\eta_{n}$, which denotes the average number of users that can be served per unit of time. When a user request arrives, it can choose between the slices that can satisfy its QoS to be served.

The delay, rate and reliability issues for users are modeled separately below.

### 4.1. Time Delay

The user’s latency is mainly composed of transmission latency, queuing latency, processing latency and frame allocation latency [9]. The transmission delay $T_{u,n}^{t}$ is the time required for user packet transmission and can be measured by the user’s packet size as well as the user’s transmission rate. In this study, it is assumed that the packets should be transmitted within one scheduling time slot, so the transmission delay is the size of one scheduling time slot. Queuing delay $W_{u,n}$ refers to the time interval from when a packet arrives in the queue buffer to when the packet is served. Processing delay $T_{u,n}^{p}$ refers to the processing delay of slices and users, which mainly depends on the processing capacity of users and slices. The frame allocation delay $T_{u,k}^{f}$ depends on the load of the network and is usually between 0 and 1 symbol length [24]. The transmission delay of the task is:

(4) $$T_{u,n}^{t}=L_{u}/B_{u}$$

where $L_{u}$ is the packet size of user *u* and $B_{u}$ is the rate assigned to user *u*.

As the computing power of the base station and the terminal increases, the processing delay is short compared to the transmission delay and can be ignored. The transmission delay and processing delay are expressed as the service delay of the service. Since the average service rate of user *u* in slice *n* is $\eta_{u,n}$, the second-order moment of the average service delay of a user *u* in slice *n* is as defined in Equation (5).

(5) $$X_{u,n}^{2}=E\left\{X_{u,n}^{2}\right\}=\frac{1}{\eta_{u,n}^{2}}$$

According to the Pollaczek–Khinchine (P-K) theorem, the average queuing delay of service packets of a user *u* for transmission on the *n*-th slice is:

(6) $$\bar{W_{u,n}}=\frac{\lambda X_{u,n}^{2}}{2(1-\rho_{u,n})}=\frac{\rho_{u,n}}{2\eta_{u,n}\left(1-\rho_{u,n}\right)}$$

where $\rho_{u,n}$ can be expressed as $\rho_{u,n}=\lambda /\eta_{u,n}$. Thus, the user’s time delay can be expressed as:

(7) $$T_{u}=T_{u.n}^{t}+W_{u,n}+T_{u,k}^{f}$$

To ensure QoS for users, the user’s latency should be no greater than the upper limit of his/her tolerable latency:

(8) $$T_{u}\leq \tau_{\max}^{}$$

where $\tau_{\max}$ denotes the upper limit of tolerable delay for slices accessed by users.

### 4.2. User Rate

The rate requested by user *u* is represented by $R_{u}$. According to Shannon’s formula, the bandwidth that can be allocated to user *u*’s access to slice *n* is:

(9) $$B_{u,n}=\frac{R_{u}}{\log(1+SINR)}$$

where $R_{u}$ denotes the transmission rate required by the user and SINR denotes the signal-to-noise ratio. When the user accesses slice *n*, according to the user’s QoS demand, slice *n* immediately allocates a certain resource $B_{u,n}$ to user *u* and ensure that the resource allocated by slice *n* does not exceed its resource limit $M_{n}$, denoted as:

(10) $$\sum_{u\in n}B_{u,n}\leq M_{n},\forall n\in N$$

### 4.3. Reliability

Since communication is mainly considered in terms of message validity and reliability, validity is related to the data rate in Section 4.2. Therefore, this subsection mainly considers the modeling of reliability. For simplicity, only reliability modeling without secondary retransmission is considered in this paper.

For the reliability requirements of the service, two main factors are considered: the failure of task completion due to the violation of the delay constraint or failure caused by the instability of the channel state [25]. According to [26], the reliability of the delay is measured by ($1-\varepsilon_{u,n}^{q}$), where $\varepsilon_{u,n}^{q}$ is the probability of delay violation, denoted as:

(11) $$\varepsilon_{u,n}^{q}=\mathrm{Pr}\left(D_{\infty }>D_{u,n}^{q,\max}\right)\approx e^{-\theta_{u,n}B_{u,n}D_{u,n}^{q,\max}}$$

where $D_{u,n}^{q,\max}$ is the upper limit of queuing delay that can guarantee the service demand, $B_{u,n}$ is the bandwidth allocated to the user, and $\theta_{u,n}$ is the QoS metric of a user *u* on slice *n*, denoted as:

(12) $$\theta_{u,n}=\ln\left(\frac{\ln(1/\varepsilon_{u,n}^{q})}{\lambda_{u}D_{u,n}^{q,\max}}+1\right)$$

We assume that the reliability threshold of the user is $\varepsilon_{u}^{g}$. To ensure the reliability requirements of the user, that is, to meet that the user’s error rate is not greater than his/her maximum tolerable error rate, the conditions that need to be met are:

(13) $$\varepsilon_{u,n}^{q}\leq \varepsilon_{u}^{g}$$

## 5. Problem Modeling

It is assumed that users accessing the slice will not leave or drop during a period of time *T*. Access control is performed with guaranteed user QoS. We denote the access of a user *u* to slice *n* by the binary variable $\alpha_{u,n}$ as

(14) $$\alpha_{u,n}=\left\{\begin{array}{c} 1,\;\mathrm{if}\;\mathrm{user}\;u\;\mathrm{access}\;\mathrm{slice}\;n\\ 0,\;\mathrm{otherwise} \end{array}\right.$$

The performance indicators of the user include data rate β, delay τ and reliability ε. The slice also has corresponding performance indicators, and when the performance indicators of the slice include the performance indicators of the user, that is, when Equation (1) is satisfied, it means that the slice can meet the QoS demand of the user, and the slice can be used as one of the access choices of the user at that time. The QoS index interval of the slice is expressed as follows:

(15) $$\left\{\left(\beta_{\min},\beta_{\max}\right),\left(\tau_{\min},\tau_{\max}\right),\left(\varepsilon_{\min},\varepsilon_{\max}\right)\right\}$$

Since only three indices of data rate β, delay τ and reliability ε are considered in this paper, the QoS metric is a three-dimensional matrix of U×3×3 in a network of *U* users. The performance index of user *u* can be described as:

(16) $$QoS_{u}=\left\{\begin{array}{c} f\left(\beta_{u},\tau_{u},\alpha_{u}\right)|\beta_{u}\in \left(\beta_{\min},\beta_{\max}\right),\\ \tau_{u}\in \left(\tau_{\min},\tau_{\max}\right),\\ \varepsilon_{u}\in \left(\varepsilon_{\min},\varepsilon_{\max}\right) \end{array}\right\}$$

All slices that can satisfy Equation (17) can be used as one of the access options for users. At the same time, to ensure the load balancing of slices, access control selection needs to be based on the remaining available resource status of slices when access control is performed.

Assume that the total resources of a slice remain constant over its lifetime. Denote the total bandwidth of slice *n* as $M_{n}$. Describe the remaining resource state of slice *n* at moment *t* as the current state of the slice based on the queue situation of the slice and the current resource occupation:

(17) $$\gamma_{n}=\frac{\sum_{u}B_{u}}{M_{n}}$$

where $\sum_{u}B_{u}$ denotes the bandwidth resource occupied by slice *n* at the current moment.

Based on the current degree of resource utilization of slices and the QoS requirements of users, access control is applied to users, and as many users as possible are provided access by optimizing the access control policy π. The number of users provided access in the system is the sum of binary variables for each user in the system as defined in Equation (18).

(18) $$A_{total}=\sum_{u\in U}\sum_{n\in N}\alpha_{u,n}$$

Considering that the resources of each slice are limited, to ensure that more users are provided access, it is necessary to allocate the minimum bandwidth $B_{u}$ that can satisfy the QoS demand of users after they are provided access to the slice so that more resources are left to provide access to more users. Therefore, the objective function is expressed as maximizing the number of users $A_{total}$ as

(19) $$\begin{array}{l} \max_{\pi ,B_{u}}\;A_{total}\\ s.t.c1:\gamma_{u}\geq \gamma^{thr},\forall u\in U\\ c2:T_{u}\leq \tau_{\max},\forall u\in U\\ c3:\varepsilon_{u,n}^{}\leq \varepsilon_{u}^{g},\forall u\in U,\forall n\in N\\ c4:\sum_{u\in U}B_{u}\leq M_{n},\forall u\in U,\forall n\in N\\ c5:\alpha_{u,n}\in \left\{0,1\right\} \end{array}$$

where the constraint *c*1 indicates that a user can be provided access when the remaining available resources of the slice can satisfy the user’s QoS request, *c*2 means that slice *n* can satisfy the delay requirement of the user, *c*3 represents that the slice can satisfy the reliability requirement of user *u*, *c*4 denotes that the sum of the bandwidth allocated to all users cannot exceed the total bandwidth of slice *n*, and *c*5 is a constraint on the access variables to ensure that a user *u* can access at most one slice at a time.

## 6. Methodology

Considering that users have multiple choices of slice access but that only one slice can be associated with a user in the end, a suitable user access policy is needed to ensure the maximum number of users that can be provided access in the system. Meanwhile, the load of the slices is relatively balanced. In this section, a preference-based access control policy is considered. First, the set of users preferred by a slice is selected based on the load of the slice. Secondly, the set of slices from which users are provided access is selected based on their QoS requirements. Finally, a set of user slices is selected for access.

### 6.1. User Multi-Access Inference

According to Table 1, the following inferences can be drawn: requests in the network have different requirements for bandwidth, latency and reliability and can be sliced separately for eMBB services (e.g., video conferencing) and URLLC services (e.g., autonomous driving), but for applications such as e-commerce and partial immersion, both eMBB and URLLC slices can be accessed. Such users who have multiple access options are defined as fuzzy users in the slice scenario, but each user can eventually be served by only one slice. Therefore, access control for fuzzy users is required.

### 6.2. Time Delay

In the scenario considered in this paper, it is difficult to decide whether a certain user request belongs to a definite type of slice and not another type because it belongs to a certain type of slice to some extent; that is, each type of task request belongs to one of the fuzzy subsets in the slice set. There exists a large number of fuzzy users and a small number of other users. Other users do not need to make access control decisions because they have only one access choice. Therefore, the set of fuzzy users that need to make access decisions should be selected first.

The set of users U={1,2,…,u} is a finite set, and the *u* users are classified into *n* classes, which are represented by the fuzzy matrix of n×u as:

(20) $$\begin{array}{c} X=\left[\begin{array}{c} x_{11},x_{12},\cdots ,x_{1u}\\ x_{21},x_{22},\cdots ,x_{2u}\\ \cdots \\ \cdots \\ x_{n1},x_{n2},\cdots ,x_{nu} \end{array}\right] \end{array}$$

where $x_{nu}$ denotes the degree of affiliation of user *u* to different slice types *n*, which is calculated by the affiliation function $F_{n}\left(x\right)$. Since this paper considers users with three QoS attributes, the affiliation function is expressed as:

(21) $$F_{n}\left(x\right)=\frac{F_{n}\left(\beta_{u}\right)+F_{n}\left(\tau_{u}\right)+F_{n}\left(\varepsilon_{u}\right)}{3}$$

where $F_{n}\left(\beta_{u}\right),F_{n}\left(\tau_{u}\right),F_{n}\left(\varepsilon_{u}\right)$ denote the affiliation functions of data rate, delay and reliability, respectively, of user *u* to slice *n*. Their affiliation functions can be calculated by Equations (22)–(24), respectively.

(22) $$F_{n}\left(\beta_{u}\right)=\left\{\begin{array}{c} \;0,\beta_{u}\leq \beta_{{}_{\min}}^{n}\cup \beta_{u}\geq \beta_{{}_{\max}}^{n}\\ \frac{\beta_{u}-\beta_{{}_{\min}}^{n}}{\beta_{{}_{\max}}^{n}-\beta_{{}_{\min}}^{n}},\beta_{{}_{\min}}^{n}<\beta_{u}<\beta_{{}_{\max}}^{n} \end{array}\right.$$

(23) $$F_{n}\left(\tau_{u}\right)=\left\{\begin{array}{c} \;0,\tau_{u}\leq \tau_{{}_{\min}}^{n}\cup \tau_{u}\geq \tau_{{}_{\max}}^{n}\\ \frac{\tau_{u}-\tau_{{}_{\min}}^{n}}{\tau_{{}_{\max}}^{n}-\tau_{{}_{\min}}^{n}},\tau_{{}_{\min}}^{n}<\tau_{u}<\tau_{{}_{\max}}^{n} \end{array}\right.$$

(24) $$F_{n}\left(\varepsilon_{u}\right)=\left\{\begin{array}{c} \;0,\varepsilon_{u}\leq \varepsilon_{{}_{\min}}^{n}\cup \varepsilon_{u}\geq \varepsilon_{{}_{\max}}^{n}\\ \frac{\varepsilon_{u}-\varepsilon_{{}_{\min}}^{n}}{\varepsilon_{{}_{\max}}^{n}-\varepsilon_{{}_{\min}}^{n}},\varepsilon_{{}_{\min}}^{n}<\varepsilon_{u}<\varepsilon_{{}_{\max}}^{n} \end{array}\right.$$

By calculating the affiliation functions $F_{n}\left(\beta_{u}\right),F_{n}\left(\tau_{u}\right),F_{n}\left(\varepsilon_{u}\right)$, we get a four-dimensional matrix of affiliation functions of U×U×U×U. For user *u*, his/her three corresponding affiliation functions are not zero, which means that user *u* has affiliation degree $F_{n}\left(x\right)$ to slice *n*. For each column of the fuzzy matrix *X*, if there is only one non-zero value, it means that the user is not a fuzzy user. Conversely, if there is more than one non-zero value, it implies that the user is a fuzzy user, and thus, the fuzzy user is filtered out.

### 6.3. Slicing Preference

In order to provide access to more users, according to the bandwidth required by users, slices are more inclined to provide access to users with small bandwidth requirements. However, according to Equation (9), the actual bandwidth allocated to a user is related to the user’s requested rate and channel conditions [29]. When a user has poor channel conditions, more bandwidth resources need to be consumed to compensate to meet his/her rate requirements. This affects the access of users with better channel conditions. Therefore, the preference for slicing is related to the bandwidth consumed by the user. The preference matrix of slicing can be expressed as a one-dimensional matrix $\Phi^{\prime }$:

(25) $$\Phi^{\prime }=\left[\varphi_{1},\varphi_{2},\ldots ,\varphi_{u}\right]$$

where $\varphi_{u}$ denotes the degree of preference of slice *n* over user *u* and $\varphi_{u-1}>\varphi_{u}$ denotes the degree of preference of slice *n* of user u−1 over user *u*.

Since the preference for slicing is related to the bandwidth consumed by the access user, the one-dimensional preference matrix $\Phi^{\prime }$ can be ranked according to the bandwidth, i.e.,

(26) $$\varphi_{u}∼\underset{u\in \{u,\ldots U\}}{\arg\;\min}B_{u}$$

Equation (26) indicates that the user with the least bandwidth required from the *u*-th user to the *U*-th user is selected and ranked in position *n* in the preference matrix of slice $\varphi_{u}$.

### 6.4. User Preference

Denote the multiple access selection policies of a user *u* as ${\mathrm{ff}}_{u}=\left(\alpha_{u,1},,\alpha_{u,2},\ldots ,\alpha_{u,n}\right)$. Consider the acceptance or rejection of user requests in different remaining available resource states of slices as a binary variable $\alpha_{u,n}$. Since the user can only access one of the slices, $\left|\right|{\mathrm{ff}}_{u}\left|\right|\leq 1$. However, the users’ preferences for different slices are different, and we denote the preference matrix of user *u* as $\Phi {}^{\prime \prime }$:

(27) $$\Phi {}^{\prime \prime }=\left[\Phi_{1},\Phi_{2},\ldots ,\Phi_{N}\right]$$

where $\Phi_{n}$ indicates the degree of preference of user *u* for slice *n*. The higher the value is, the higher the preference.

Each user attribute corresponds to a set of acceptable slice resource states, and the remaining resource state is denoted as $\Upsilon =\{\gamma_{1},\gamma_{2},\ldots ,\gamma_{n}\}$. Under different slice states, user preferences for slices differ according to the access policy π and are related to the user’s QoS requirements and the remaining available resource state of the slice:

(28) $$\begin{array}{c} \Phi_{n}=\left[\Phi_{1},\Phi_{2},\ldots ,\Phi_{\Upsilon },\right]\\ =\left[\begin{array}{c} \varphi_{1,1},\varphi_{1,2},\ldots ,\varphi_{1,\Upsilon }\\ \varphi_{2,1},\varphi_{2,2},\ldots ,\varphi_{2,\Upsilon }\\ \ldots \\ \ldots \\ \ldots \\ \varphi_{N,1},\varphi_{N,2},\ldots ,\varphi_{N,\Upsilon } \end{array}\right] \end{array}$$

where column *i* indicates the preference of user *u* for different slices when the remaining resources of the slice are in state $\gamma_{i}$. Row *n* indicates the degree of preference of user *u* for slice *n* in different resource states. Equation (28) indicates that the user selects the slice that can satisfy his/her QoS according to the attributes of the slice, and if the resource state of the slice cannot satisfy the QoS demand, then the user’s preference degree $\varphi_{n,\gamma }=0$ for slice *n*.

The user’s preference for slices is related to the affiliation degree $F_{n}\left(x\right)$ between the user’s QoS requirements and the QoS attributes of slices, and the larger the affiliation degree, the higher the value of the user’s preference for slices. However, the degree of affiliation is only the preference for QoS attributes, and the remaining resource state of the slice should be fully considered to ensure the QoS requirements of users. The preference degree $\varphi_{n,\gamma }$ of user *u* for slice *n* as a whole is expressed as:

(29) $$\left\{\begin{array}{c} \varphi_{n,\gamma }∼F_{n}\left(x\right),\;\;\mathrm{Remaining}\;\mathrm{resource}\;\mathrm{of}\;\mathrm{slice}\;n\;\mathrm{meets}\;\mathrm{user}\;\mathrm{QoS}\;\\ \varphi_{n,\gamma }=0,\;\;\mathrm{Otherwise}\; \end{array}\right.$$

Equation (29) indicates that users have a corresponding preference value for slices that can satisfy their QoS needs. It implies that user *u* prefers slices with a QoS similar to his/her demand, but as the user is provided access, the preference value changes to 0 when the remaining resources of the slice are insufficient to satisfy his/her QoS. The preference value can be updated by calculating the user’s preference value for each slice and the remaining resource status of each slice.

### 6.5. Slice–User Association Strategy

Based on the slice preferences and user preferences, a set of slice–user association schemes are designed. There are multiple slices preferring the same user in this association scheme, and the final slice–user association decision is related to the remaining resource status of the slice itself, considering the load balancing of the achieved slices. The resource utilization status of the slice is used as the criterion for user access and is denoted as:

(30) $$\mu =\min\{\gamma_{1},\ldots ,\gamma_{n}\}$$

In the case of multiple slices associated with the same user, Equation (30) indicates that the slice with the lowest resource utilization among them is selected for user access.

## 7. Simulation Analysis

In this paper, we use the Python 3.7 simulation platform to validate the preference-aware user access control policy. A C-RAN system consisting of one RRH, two network slices, one BBU pool and an optical forwarding network is considered, and 100 users with different QoS requirements are randomly generated. We compare our results with the QoS-guaranteed user access control strategy [30], greedy-based access control strategy and random-based access control strategy to maximize the number of users accessed. The simulation parameters are shown in Table 2.

**Figure 3:**
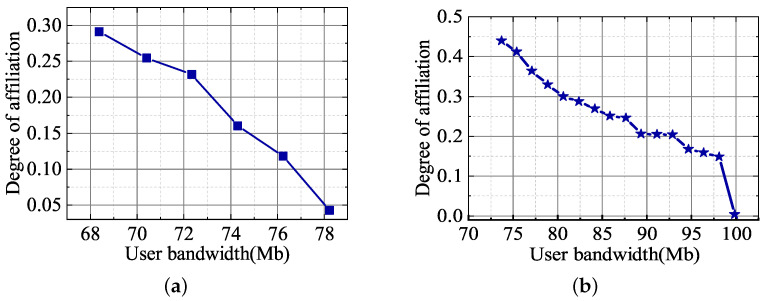
Degree of affiliation: (**a**) Slice 1 and (**b**) Slice 2.

Figure 3 shows the user’s degree of affiliation to different slices. The horizontal axis indicates the bandwidth interval of the slice, and the vertical axis indicates the user’s degree of affiliation. It can be seen that the subscriber’s affiliation level decreases as the required bandwidth approaches the upper limit that can be satisfied by the slice. This is because users prefer slices that can provide higher bandwidth to ensure their QoS is satisfied. For the overlapping area of the bandwidth of Slice 1 and Slice 2 in the figure, it can be seen that users have different preferences for the same bandwidth because the lower limit of bandwidth that can be provided by different slices is different, which is related to the affiliation function.

**Figure 4:**
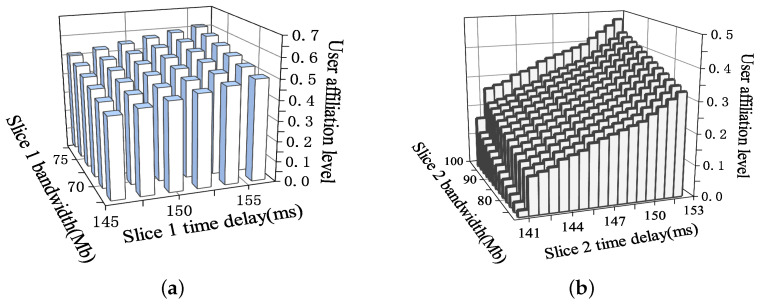
User affiliation with joint consideration of bandwidth and latency: (**a**) Slice 1 and (**b**) Slice 2.

Figure 4 shows the degree of affiliation of users with joint consideration of delay and bandwidth. It can be seen that the degree of user affiliation decreases with the increase in required bandwidth and increases with the increase in required delay. This is due to the instability of the channel state and the need to guarantee the minimum delay required for the users. Therefore, the smaller the minimum delay that can be satisfied for a slice, the higher the user’s degree of preference. Therefore, for a certain slice QoS interval, the less delay-sensitive and less bandwidth-demanding users have a higher degree of affiliation with the slice. In addition, due to the variability of QoS between slices, the degree of affiliation of the same user to different slices also varies. For example, a user with a bandwidth requirement of 78 Mb and a latency requirement of 150 ms has a joint affiliation of about 0.7 for Slice 1, while the joint affiliation for Slice 2 is only 0.56.

**Figure 5:**
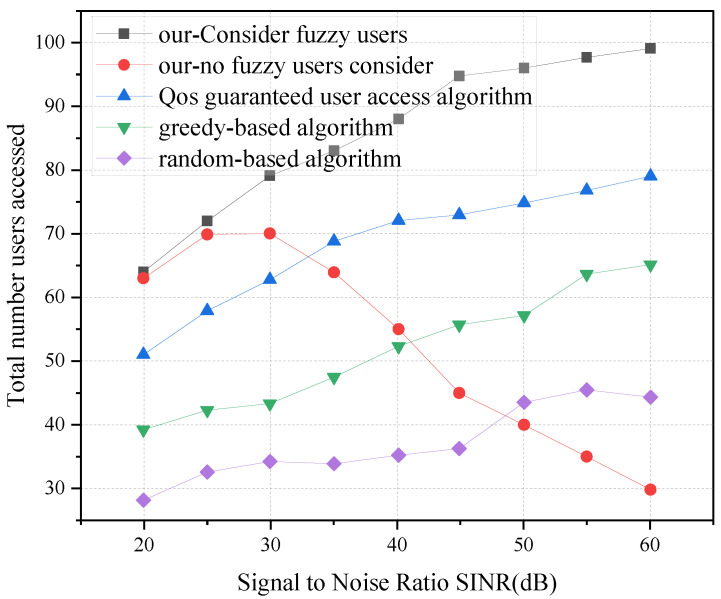
Impact of different numbers of fuzzy users on user access.

Figure 5 shows the effect of different fuzzy user percentages on the number of users provided access in the system. It can be seen that as the percentage of fuzzy users increases, more users are provided access by the proposed algorithm that considers fuzzy users, which can provide access to 20.2% more users than the QoS-guaranteed user access algorithm when the number of fuzzy users is 60% of the total users in the system. The QoS-guaranteed user access algorithm allocates more resources to users to ensure their quality of service, while the algorithm proposed in this paper allocates resources to users to satisfy their QoS to provide access to more users, so that there are more remaining resources to provide access to more users. In the algorithm that does not consider fuzzy users, the number of accessed users decreases gradually as the proportion of fuzzy users increases because fuzzy users account for a certain proportion in the QoS interval of slices, and not considering fuzzy users leads to some users not being able to access the slices, and thus, the number of users provided access decreases. We also find that the performance of our proposed algorithm that considers fuzzy users is much higher than greedy-based and random-based access control strategies.

**Figure 6:**
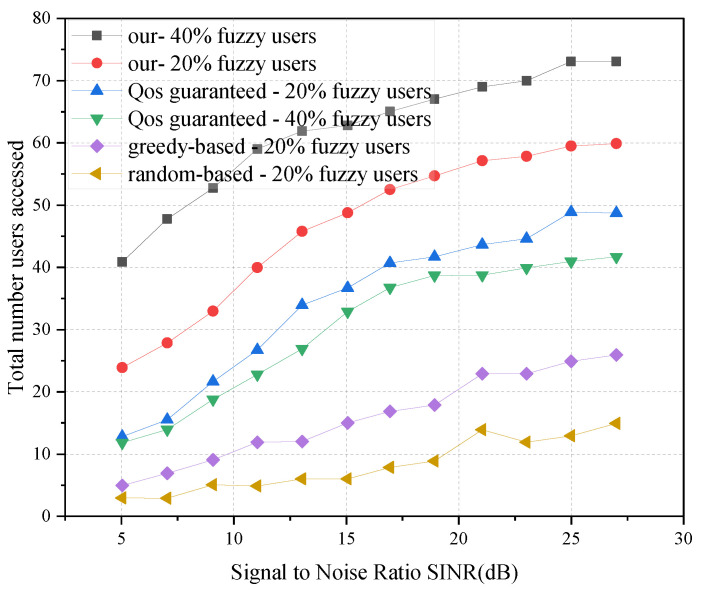
Impact of different numbers of fuzzy users on user access.

Figure 6 shows the number of subscribers who are provided access at different signal-to-noise ratios. It can be seen that as the S/N ratio increases, the overall trend of the number of users provided access increases. This is because the S/N ratio increases and users have less bandwidth to make up for channel consumption, i.e., users can use less bandwidth to meet the data rate demand. Therefore, more surplus resources can be available to provide access to more users. When considering 40% fuzzy users and a signal-to-noise ratio of 25 dB, the proposed algorithm in this paper can provide access to 42.46% more users than the QoS-guaranteed user access algorithm. It can also be seen from Figure 6 that the performance of our proposed method is better than the greedy-based and random-based access control algorithms for both 40% and 20% fuzzy users. Among them, the performance based on greedy is slightly better than that based on random.

**Figure 7:**
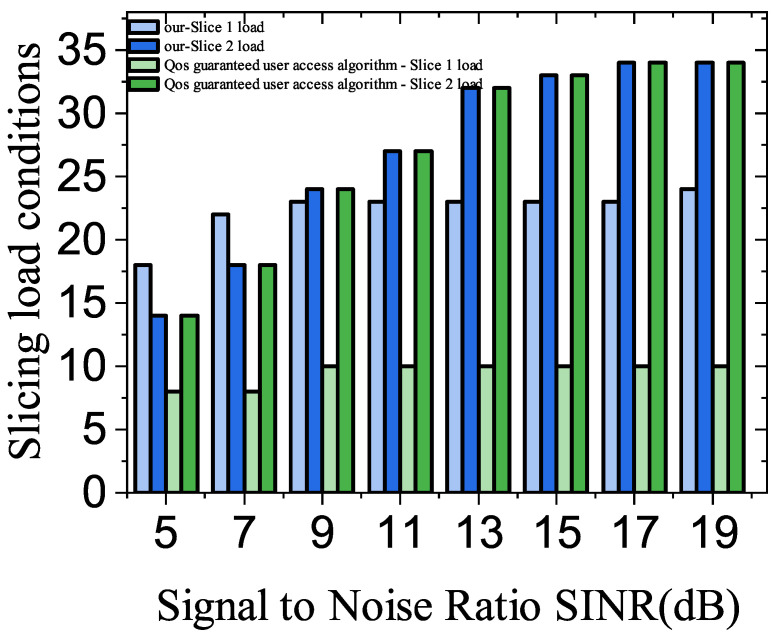
Impact of different numbers of fuzzy users on user access.

Figure 7 shows the slice loadings under different signal-to-noise ratios. In the case of a small signal-to-noise ratio, the slice load situation of the proposed algorithm is relatively balanced. At a signal-to-noise ratio of nine, the difference between the load of Slice 1 and Slice 2 in the proposed algorithm is 9.1%, while the difference between the load of Slice 1 and Slice 2 in the comparison algorithm is 50%. When the S/N ratio is greater than nine, it can be seen that the number of users provided access by Slice 1 increases slowly, which is related to the QoS requests of users in the system, indicating that the number of users that can be satisfied by Slice 1 has reached the upper limit.

## 8. Conclusions

To solve the problem of multiple access for a single service for IoT, this paper proposes a preference-aware user access control strategy. Firstly, a fuzzy user selection strategy is proposed by establishing user QoS requirements and slice QoS intervals and affiliation functions. Then, a user access control strategy is designed, and user–slice grouping is performed based on the slice preference, the user preference matrix and the load of the slice. Simulation results show that the proposed algorithm can increase the number of users who are provided access to the system while ensuring user QoS. The flexibility of network slicing and the mobility of users increases the difficulty of resource management. Traditional network models have difficulty making optimization decisions, and artificial intelligence is usually used to solve complex problems. Therefore, artificial intelligence algorithms can be applied to C-RAN network slice resource management strategies in the future to improve the accuracy and flexibility of resource management.

## Figures and Tables

**Table 1:** QoS metrics [27,28].

Business Type	Speed	PTime Delay Limit	Reliability
Video teleconferencing	High speed	50 ms	Must be reliably transmitted
E-commerce	Appropriate speed	100 ms	Must be reliably transmitted
Email	Low speed	NA	Do your best
HTML web browsing	Variable speed requirements	NA	Do your best
Autopilot	>100 Mbps	<10 ms	Must be reliably transmitted
AR	High speed	<20 ms	∖
Primary immersion	20 Mbps	>40 ms	∖
Partial immersion	100 Mbps–1 Gbps	<30 ms	∖
Deep immersion	1–4 Gps	<13 ms	∖
Total immersion	>4 Gps	<8 ms	∖

**Table 2:** Simulation parameter settings.

Parameter Settings	Parameter Values
Transmitting power	1 W
Noise power	$10^{-13}$ W
Number of users	100
Number of slices	2
Packet size	0.2–0.5 M
Average arrival rate	2000 pps
User rate interval	[40, 100] Mb/s
User time delay interval	[130, 160] ms

## Data Availability

Not applicable.
